# Special issue: advances in immunology and its applications

**DOI:** 10.3724/abbs.2025247

**Published:** 2026-01-25

**Authors:** Mingshun Han, Hongyan Wang

**Affiliations:** Key Laboratory of RNA Science and Engineering CAS Center for Excellence in Molecular Cell Science Shanghai Institute of Biochemistry and Cell Biology University of Chinese Academy of Sciences Chinese Academy of Sciences Shanghai 200031 China

Innate immunity and adaptive immunity play crucial roles in regulating inflammation-related diseases such as tumors, infections, and autoimmune diseases. The interplay between innate immunity and adaptive immunity ensures the maintenance of tissue homeostasis and effective clearance of invading pathogens or tumor cells. However, dysregulation in each type of immune cells or their communications leads to pathological conditions, ranging from chronic inflammation to malignant progression. In recent years, the field of immunology has witnessed a paradigm shift, moving from merely describing immune cell signaling or phenotypes to actively exploring strategies that reshape immune functions for therapeutic benefit.

Exploring effectors or strategies to reshape T cell function for the improved anti-tumor and anti-infection efficacy has emerged as a critical research direction. This includes the development and optimization of chimeric antigen receptor T-cell (CAR-T) therapies for clinical applications. While CAR-T therapy has revolutionized the treatment of hematologic malignancies, its success in solid tumors remains limited. Chen *et al*. [1] dissect the distinct signaling mechanisms of chimeric antigen receptors (CARs) compared to T cell receptors (TCRs). They elucidate how CAR-T cells, despite overcoming MHC restrictions, encounter significant hurdles such as inefficient tumor infiltration and the hostility of the immunosuppressive tumor microenvironment (TME). To address these challenges, the authors underscore several innovative strategies, such as optimizing receptor clustering to facilitate immune synapse formation and integrating novel co-stimulatory domains to augment therapeutic efficacy in solid tumors.

Moving beyond engineering, fundamental insights into microenvironmental stress and T cell intrinsic signaling are pivotal. While oxidative stress is a well-established concept in the TME, Ji and Xiao [2] draw attention to the phenomenon of “reductive stress”. They explain how a surplus of intracellular reducing agents disrupts redox balance, creating a reductive environment that significantly influences immune cell differentiation and tumor survival. In a complementary study, Shi *et al*. [3] investigate the intrinsic role of pattern recognition receptor (PRR) signaling within T cells. Although PRRs are historically classified as innate sensors, this review highlights their critical function in adaptive immunity, discussing how T cells utilize PRRs to interpret endogenous danger signals and microbial cues to regulate cytokine release and proliferation. Additionally, Ma *et al*. [4] provide a comprehensive update on T cell immunoglobulin and mucin-containing molecule 3 (TIM-3), an important immune checkpoint. By detailing its expression profile across T cells, NK cells, and myeloid lineages, they propose that rational combination therapies targeting TIM-3 alongside other checkpoints offer a promising avenue to surmount current resistance mechanisms.

This issue also highlights the regulation of immunity by neurotransmitters and biological rhythms, illustrating the profound integration of the immune system with physiological networks. Fan and Zhao [5] summarize the neurotransmitter-receptor landscape in T cell tumor immunology. They elaborate on how specific neurotransmitters, including glutamate, acetylcholine, GABA, and serotonin, could dictate T cell activation and differentiation within the TME. The authors advocate for targeting these neuro-immune axes, such as through β-blockers or glutamate receptor inhibitors, as a new frontier for enhancing cancer immunotherapy. From a different perspective, Sun *et al*. [6] assess the influence of circadian rhythms on the TME and immunotherapy outcomes. They present evidence indicating that the efficacy of treatments like immune checkpoint blockade depends on administration timing, suggesting that future clinical protocols should incorporate chronobiology to maximize patient benefit. Further exploring this dimension, Zhao *et al*. [7] examine the bidirectional circadian dialogue between the host and the gut microbiota. They describe how modern lifestyle factors, such as shift work and irregular dietary habits, disrupt this synchronization, resulting in compromised barrier integrity and systemic metabolic disorders.

This issue also focuses on the function of various innate immune cells, including ILCs, macrophages, and dendritic cells (DCs), as well as their crosstalk with T cells in maintaining homeostasis across lung, gut, and aging contexts. Chen *et al*. [8] concentrate on Group 2 innate lymphoid cells (ILC2s), which functionally mirror Th2 cells. They underscore the context-specific roles of ILC2s in pulmonary diseases, explaining how these cells react to environmental alarmins such as IL-33 and thymic stromal lymphopoietin (TSLP). The review also discusses targeting ILC2 plasticity as a potential therapeutic intervention for lung inflammation. Recent findings suggest lipid metabolism as a crucial determinant of innate immune function. Huang *et al*. [9] offer an updated survey of lipid-regulated immunobiology in macrophages, examining how fatty acyl composition and oxidative states drive macrophage polarization and fate changes in the context of cancer. Focusing on the intestinal environment, Wang and Liu [10] investigate the metabolic interplay between DCs and the gut microbiota. They discuss how DCs detect microbial metabolites such as short-chain fatty acids to navigate the delicate equilibrium between immune tolerance and inflammation. Furthermore, Zheng *et al*. [11] address dysregulated immunometabolism in gut inflammation, exploring how metabolic reprogramming—such as impaired glycolysis or fatty acid oxidation—affects immune cell differentiation in conditions such as inflammatory bowel disease (IBD).

Aging is closely related to immune function. Shen *et al*. [12] provide the possible mechanisms to understand intestinal “immunosenescence” in the face of an aging population. They analyze the link between immune aging and microbiota dysbiosis in driving chronic inflammation and neurodegenerative diseases, suggesting that dietary and microecological interventions might be instrumental in extending the healthspan of the elderly.

Special thanks are extended to all authors for their contributions, who not only summarize existing knowledge but also offer insightful hypotheses and identify future challenges. These reviews will help us to learn the cutting-edge progress in immunology and its potential application to various kinds of diseases. We hope this special issue serves as a valuable resource for researchers and clinicians, paving the way for the next generation of immunological breakthroughs.
